# Letter to the Editor: Trends in coronary artery disease mortality among hyperlipidemic patients: Geographic, gender, and racial insights from CDC WONDER data (1999–2020)

**DOI:** 10.1016/j.ijcrp.2026.200581

**Published:** 2026-01-20

**Authors:** Mohamed Fawzi Hemida

**Affiliations:** Alexandria Main University Hospital, Alexandria, Egypt

Dear Editor,

We read with great interest the recently published article by Naveed et al. examining coronary artery disease (CAD) mortality trends among patients with hyperlipidemia using the CDC WONDER database [1]. While the topic is timely and of significant public health relevance, we would like to respectfully raise a critical methodological concern regarding the ICD-10 coding strategy used to define hyperlipidemia, which may have important implications for the validity of the study's findings.

In the Methods section, the authors report using the ICD-10 code E78 to identify decedents with hyperlipidemia. However, according to the official ICD-10-CM classification system maintained by the Centers for Disease Control and Prevention (CDC), E78 is a non-billable parent category titled “Disorders of lipoprotein metabolism and other lipidemias” and includes multiple distinct conditions. Specifically, hyperlipidemia is defined by the subcodes E78.0 (pure hypercholesterolemia), E78.1 (pure hyperglyceridemia), E78.2 (mixed hyperlipidemia), E78.3 (hyperchylomicronemia), E78.4 (other hyperlipidemia), and E78.5 (hyperlipidemia, unspecified). In contrast, the E78 category also includes non-hyperlipidemia lipid disorders, such as E78.6 (lipoprotein deficiency), E78.8 (other disorders of lipoprotein metabolism), and E78.9 (disorder of lipoprotein metabolism, unspecified). Accordingly, use of the umbrella code E78 alone does not accurately define hyperlipidemia and may result in inclusion of heterogeneous lipid metabolism disorders, leading to misclassification bias [2].

Accurate use of hyperlipidemia-specific subcodes is essential in epidemiologic analyses using administrative mortality data to ensure diagnostic specificity and valid inference. Notably, a recent national retrospective cohort study by Pham et al. explicitly defined hyperlipidemia using ICD-10-CM codes E78.0–E78.5 when evaluating cardiovascular mortality outcomes, thereby adhering to established coding standards and best practices [3]. In contrast, reliance on the broader E78 category may inadvertently inflate case counts and alter age-adjusted mortality rates, temporal trends, subgroup analyses, and geographic comparisons.

Importantly, redefining hyperlipidemia using the appropriate ICD-10-CM subcodes (E78.0–E78.5) would be expected to yield materially different results, particularly with respect to absolute mortality estimates and observed demographic and geographic disparities. As such, the reported outcomes, interpretations, and conclusions of the current study may not accurately reflect CAD mortality specifically attributable to hyperlipidemia. This issue extends beyond a minor coding distinction and directly affects the internal validity, interpretability, and reproducibility of the analysis.

We believe that clarification of the coding approach or reanalysis using the correct ICD-10-CM subcodes would substantially strengthen the study and enhance confidence in its conclusions. We offer these comments in the spirit of constructive scientific discourse and with appreciation for the authors’ contribution to this important area of cardiovascular epidemiology.

## Ethics statement

As this submission is a Letter to the Editor, ethical approval was not applicable.

## Funding

The authors received no financial support for the research, authorship, or publication of this manuscript.

## Declaration of competing interest

The authors report no conflicts of interest, financial or otherwise, related to this work.

## Data Availability

No original datasets were generated or analyzed for this Letter to the Editor. The discussion is based on publicly available mortality data from the CDC WONDER database, referencing ICD-10-CM codes for hyperlipidemia (E78.0–E78.5) and coronary artery disease (I20–I25), as cited in the published literature.
